# Insomnia in seasonal affective disorder: considering the use of benzodiazepines with a focus on lormetazepam

**DOI:** 10.3389/fpsyt.2025.1667086

**Published:** 2026-01-14

**Authors:** Giovanni Biggio, Claudio Mencacci

**Affiliations:** 1 Department of Life and Environmental Sciences, University of Cagliari, Cittadella Universitaria di Monserrato, Cagliari, Italy; 2 Institute of Neurosciences, National Research Council (C.N.R.), Cittadella Universitaria di Monserrato, Cagliari, Italy; 3 Department of Neuroscience and Mental Health, Azienda Socio Sanitaria Territoriale (ASST) Fatebenefratelli Sacco, Milan, Italy

**Keywords:** benzodiazepines, depression, insomnia, intermittent dosing, lormetazepam, seasonal affective disorder, sleep

## Abstract

**Background:**

Seasonal affective disorder (SAD) occurs in two main forms: winter-pattern SAD, associated with depressive symptoms during shorter, darker days; and summer-pattern SAD, linked to mood disturbances during longer, hotter days. SAD may develop into a chronic condition with recurring depressive episodes. Risk factors for SAD include geographic latitude, age, gender, genetic predisposition, and lifestyle. Sleep disturbances, such as insomnia, hypersomnia, and circadian rhythm disruptions, are common and can amplify emotional symptoms.

**Objective:**

This review explores the clinical features and management strategies for insomnia associated with SAD, focusing on the potential of benzodiazepines (BZDs), in particular lormetazepam.

**Results:**

Controversies surround current nonpharmacological and pharmacological strategies for managing sleep disorders in SAD. This review emphasizes the importance of using more effective treatments for insomnia associated with SAD, currently an unmet need. In particular, clinical evidence supports the potential benefits of intermittent hypnotic BZDs to treat insomnia. Among the BZDs, short-term or intermittent use of lormetazepam is an effective treatment option in the management of insomnia.

**Conclusion:**

Insomnia associated with SAD is an important symptom to monitor because it impacts the patient’s quality of life. BZDs, including lormetazepam, are a standard short-treatment option for insomnia that could improve the sleep symptoms associated with SAD. Comparative clinical trials of the efficacy and safety of lormetazepam in this patient population are required to confirm this.

## 1 Introduction

Sleep disturbances are a common symptom of seasonal affective disorder (SAD) (1, 2) and may potentially amplify the mood symptoms of SAD. Benzodiazepines (BZDs) and BZD-like drugs (the ‘Z-drugs’) are effective hypnotics and are widely prescribed for chronic insomnia (3). As such, their use should be considered for patients with insomnia associated with SAD. Lormetazepam is a BDZ with a well-established clinical efficacy and, like other drugs of this class, can potentially be used in a cyclical or intermittent regimen (4, 5). Due to its pharmacokinetic and pharmacodynamic characteristics is therefore particularly appropriate for a disorder such as SAD that has a seasonal course. In this review, we provide a brief overview of SAD followed by a more detailed discussion of insomnia in SAD and its management, with a focus on BDZs, and more specifically, lormetazepam.

## 2 An overview of SAD

The seasonal nature of mood disorders has been acknowledged since antiquity, with Hippocrates the first to describe the important link between seasons and mood changes. He believed that the proper study of medicine should first observe the development of the seasons (as described in his work ‘On Airs, Waters, and Places’). Chronobiological concepts link circadian rhythm disturbances to the seasonality of SAD (6), which has recently been acknowledged to have a distinct nosographic autonomy (7, 8). This mood disorder, specifically its depressive component, is closely linked to the periods of the year and to the time of light exposure; recurrent winter episodes alternate with periods of euthymia or hyperthymia in spring−summer (winter SAD) (9), while a summer disorder (summer SAD) shows clinical manifestations between May and September (Northern Hemisphere) and hypomanic phases in winter (10). Evidence suggests that winter SAD may be triggered in susceptible individuals by the reduced ambient light that occurs in winter, whereas summer SAD may be triggered by high temperatures (11).

Key risk factors for SAD include geographic latitude, age, gender, genetic predisposition, and lifestyle, which are discussed below.

Estimated worldwide prevalence rates of SAD vary between 1% and 10% of the population, depending on geographic location; regions further from the equator, which receive less sunlight in winter and more in summer, tend to have higher rates of SAD (1, 12). As a result, SAD is more common in northern populations, correlating with latitude. The prevalence of SAD is also affected by age. Although SAD can affect children, it tends to start around the time of puberty, worsening during adolescence, and becoming more severe in the third to fourth decade of life (13). The prevalence of SAD tends to be higher among women than men (1, 12). In Italy, the prevalence of SAD is estimated to be between 3.5% and 11.5% for the subsyndromal form (S-SAD), more commonly known as “winter blues”, and the ratio of women to men with SAD is about 3:1 (14).

There is a close association between depression and menopause (15). Menopausal women with SAD typically experience autumn/winter symptoms, including subjective dysphoria, sleep disturbance, severe asthenia, increased appetite, and carbohydrate cravings (13).

The seasonal pattern is added to the diagnosis in patients with recurrent affective disorders (unipolar major depression or bipolar I or II disorder) with seasonal depressive relapses (16). In addition, genetic predisposition can increase the risk of SAD (1, 17). Indeed, seasonal changes in mood and behavior tend to run in families, especially winter SAD. This is largely due to a biological predisposition that contributes at least 29% to the onset of SAD (11).

From a biological viewpoint, people with SAD have a dysregulated serotonergic system, which is one of the factors responsible for mood balance. Patients with SAD have shown seasonal fluctuations in binding to the serotonin transporter (SERT) protein in the brain (18). The SERT protein is responsible for transporting serotonin from the synaptic cleft to the presynaptic neuron. Thus, higher levels of SERT lead to lower levels of serotonin activity (19). SERT levels are kept low by sunlight throughout the summer, but they rise in the winter, causing depression and SAD (19).

In addition, patients with SAD may experience overproduction of melatonin, a hormone produced by the pineal gland that facilitates sleepiness and normalizes the circadian rhythm during hours of darkness. In healthy people with normal sleep-wake cycles, the physiological production of melatonin usually begins in the evening, 14 hours after spontaneous wakening (20). As winter days become darker, melatonin production increases, inducing sleepiness and lethargy in patients with SAD (19). The combination of reduced serotonin and increased melatonin disrupts the circadian rhythm (19).

Gamma−aminobutyric acid (GABA)-mediated signaling within the suprachiasmatic nucleus (SCN, the ‘master circadian clock’), is implicated in regulating the seasonal rhythm in animal models and the circadian rhythm in mammals (21, 22). However, seasonal variations in GABA levels in various brain regions implicated in the pathophysiology of SAD such as the putamen and insula (but not the SCN) could not be verified in healthy human volunteers in a recent study (22). Since there was no seasonal variation in GABA content in these brain regions, the study authors suggested future investigations should determine whether there are seasonal variations in GABA levels in patients with SAD, and whether there is seasonal variation in GABAergic neurotransmission attributable to changes in GABA receptor functioning (22).

## 3 Symptoms of SAD: a focus on sleep

SAD can become a chronic condition with cyclical depressive episodes. Winter and summer SAD are characterized by a lack of energy, a state of sadness, and reduced sociability (11). The most common symptoms of SAD include hypersomnia or insomnia, hyperphagia or hypophagia, mental fatigue, asthenia, concentration difficulties, and irritability (1). A specific feature of SAD is the presence of a depressed but reactive mood, which allows a significant change in mood (accentuated in the evening hours) following a positive event.

Symptoms starting in autumn and winter may include tiredness, fatigue, mood deflection, irritability, difficulty concentrating, musculoskeletal pain, decreased sexual desire, hypersomnia, increased appetite, and weight gain (10, 13). The acute phase usually occurs during the darker months, and, in severe cases, SAD may be associated with thoughts of suicide (19). Notably, winter SAD is frequently associated with an initial hypersomnia that is often underestimated, which can evolve into forms of secondary insomnia (2).

The most common symptoms of summer SAD are insomnia, loss of appetite, weight loss, irritability, difficulty concentrating, anxiety, psychomotor agitation, and mood swings (10).

Sleep disturbances, such as insomnia, are among the most common symptoms of SAD (2). In healthy individuals, light synchronizes the circadian clock with the Earth’s solar day and has downstream effects on sleep, alertness, and mood (2). Seasonality and light therapy suggest SAD is related to abnormal responses to low light levels in winter. Specifically, SAD is characterized by a delay in the circadian phase relative to the sleep-wake cycle (circadian misalignment); delayed circadian timing is thought to manifest behaviorally as early-onset insomnia and morning hypersomnolence, with difficulty falling asleep and waking up the next morning (23). Poor sleep quality associated with SAD manifests as fragmented sleep due to frequent awakenings during the night (2). Hypersomnolence is an atypical vegetative symptom of depression and is considered a cardinal symptom in the characterization and diagnosis of SAD, expressed as either excessive daytime sleepiness and/or increased sleep duration (2). A registry study based on data from clinical interviews reported that individuals with hypersomnia in SAD sleep 72 minutes longer in winter than summer (24).

Diagnostic criteria for chronic insomnia disorder note that it compromises the patient’s lifestyle, causes cognitive decline with a reduction of executive processes, loss of occupational productivity, and potential physical damage due to fatigue-related accidents (25). Insomnia is also considered to be an independent risk factor for cardiovascular and neurological disorders, as well as psychiatric comorbidities, increasing suicidal risk (25, 26). Of concern, many individuals with insomnia inappropriately self-medicate instead of seeking formal care, and Italy has a high rate of sedative-hypnotic misuse (26). Historically, insomnia was viewed as primarily a symptom associated with somatic or mental disorders, but the latest European Insomnia Guideline 2023 classifies insomnia as an independent disorder (25). Nevertheless, insomnia tends to be under-recognized in Italy, where most primary care physicians continue to consider insomnia to be a comorbid condition (26). Given the high prevalence of insomnia symptoms and their serious health implications, as well as its under-diagnosis, the recognition, diagnosis, and adequate treatment of insomnia represents an important unmet need.

## 4 Nonpharmacological management of sleep disorders in SAD

Treating sleep disorders is crucial to alleviating the overall symptoms of SAD and improving quality of life. Bright light therapy (BLT) is considered a first-line treatment for SAD, despite the fact that while some studies have shown effectiveness, others have not (27). Exposure to bright light in the morning aims to redress the serotonin-melatonin balance to regulate the circadian sleep-wake rhythms and have an effect on both mood and sleep (28). Lifestyle modification offers a wide range of additional intervention options. Accordingly, some studies have shown a beneficial effect of exercise, with or without BLT, combined with sleep hygiene rules, dietary changes and the integration of supplements (including vitamin D), and melatonin (19, 29). Psychological support based on cognitive behavioral therapy (CBT) can help to manage negative thoughts about sleep and SAD (19). However, the use of CBT requires qualified personnel and is not widely available in all countries (30). Moreover, evidence-based insomnia management guidelines are not routinely used in mental healthcare (31). The emergence of fully-automated digital CBT (dCBT) may transform the insomnia treatment landscape, as this treatment delivery format can be efficiently integrated into both population health and clinical health service initiatives, optimizing treatment for people across all levels of complexity and need (30). A real advantage of dCBT products is that they could potentially deliver clinical guideline care for insomnia at a population scale (30).

## 5 Pharmacological treatment options for insomnia

**Figure 1:**
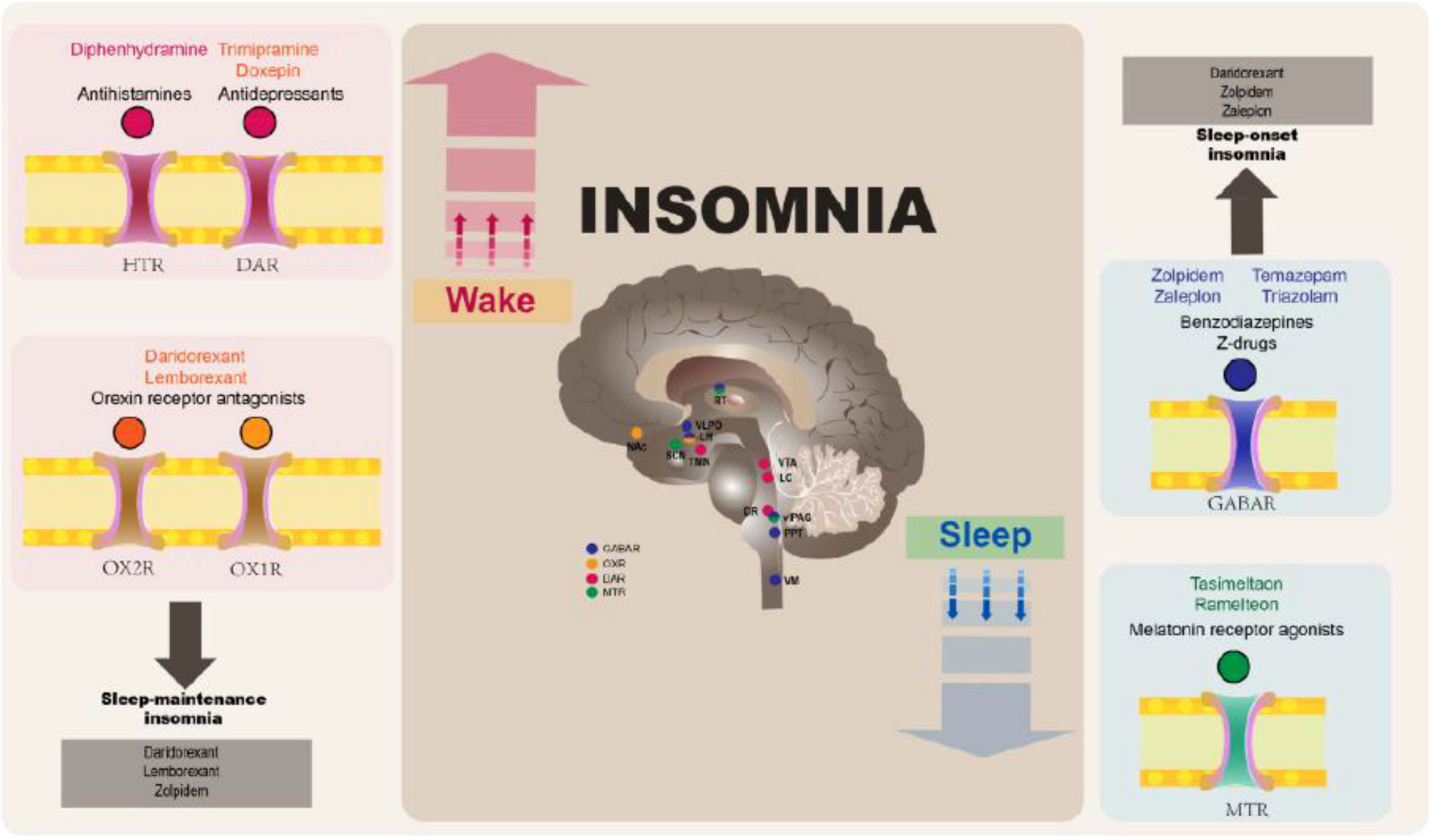
The mechanism of action of different insomnia medications. DAR, dopamine receptor; GABAR, gamma−aminobutyric acid receptor; HTR, histamine receptor; MTR, melatonin receptor; OXR, orexin receptor. Source: Reproduced from Yue JL, Chang XW et al. Efficacy and tolerability of pharmacological treatments for insomnia in adults: A systematic review and network meta-analysis. Sleep Med Rev (2023) 68:101746. Doi: 10.1016/j.smrv.2023.101746 (32), originally published by and used with permission from Elsevier.

Pharmacological treatments target the brain’s neurotransmitter systems and neurochemistry that regulate the sleep-wake cycle (**Figure 1**). Key neurotransmitters that regulate sleep and wakefulness include GABA, acetylcholine, and orexin (27): the binding of GABA to its receptor opens an ion channel that allows chloride ions to move into the cell and thereby exert a hyperpolarizing (inhibitory) function (33) which, in the late evening, facilitates the induction of non-rapid eye movement (NREM) sleep. The knowledge of neurochemical changes associated with NREM and rapid eye movement (REM) sleep, as well as those associated with awakening, together with an understanding of how pharmacotherapies may affect these systems, is crucial to help clinicians choose the most appropriate insomnia medications.

Hypnotic BZDs and the Z-drugs are the most widely used drugs prescribed for chronic insomnia (3), and are discussed in more detail in Sections 5.1 and 5.3. Importantly, these classes of molecules differ from each other, both in terms of their effects on the sleep and in terms of dosage and administration regimens.

More recently, dual orexin receptor antagonists (DORAs) have been approved to manage chronic insomnia in patients with a range of psychiatric disorders. DORAs reduce wakefulness by inhibiting the effect of orexin through binding to OX1 and OX2 receptors localized on monoaminergic and cholinergic neurons of different brain areas (34). Since activation of OX neurons by orexin also leads to activation of GABAergic neurons in the ventral hypothalamic preoptic area and consequently inhibition of sleep-promoting GABA/galanin-containing neurons in the same area (35), it could be hypothesized that the effect of DORAs is also mediated via this latter polysynaptic circuit. Some examples include suvorexant, lemborexant, and daridorexant, which can be used for ≥3 months and up to 1 year for chronic insomnia (34, 36, 37).

Melatonin receptor agonists (MRAs) including melatonin and ramelteon have been investigated for their effectiveness in treating insomnia, but results from two network meta-analyses were inconsistent (32, 38). Targeting the melatonergic pathway remains an area of active investigation however. Agomelatine is a melatonin MT_1_ and MT_2_ receptor agonist and neutral antagonist at the serotonin 5-HT_2C_ receptor usually used in patients with major depressive disorder (39). A recent proof-of-concept 24-week study of agomelatine in combination with CBT in patients with moderate to severe insomnia reported encouraging reductions in insomnia severity, whereas reductions in efficacy over the duration of the study were observed with the active comparator clonazepam (40).

Drugs such as antidepressants, antihistamines, and antipsychotics are often prescribed off-label, in particular the antidepressants (e.g., mirtazapine, trazodone), for their effective sedative action to patients with insomnia (25, 41, 42). However, the European Sleep Research Society 2023 guidelines recommend very careful use of antidepressants and antipsychotics to treat insomnia due to their contraindications and adverse effects (25).

Further, antipsychotic medications (e.g., quetiapine, olanzapine) are not recommended for the treatment of insomnia due to the lack of randomized controlled trial evidence on the use of these substances concerning insomnia disorder with or without comorbidities (25).

Insufficient evidence supports the use of antihistaminergic drugs in the short- or long-term treatment of insomnia (25). Not only are there no high-quality randomized placebo-controlled clinical studies, but these drugs are known to induce a marked sedative, not hypnotic, effect associated with a rapid development of tolerance (25).

### 5.1 Benzodiazepines

BZDs are positive allosteric modulators of GABA_A_ receptors in the central nervous system (CNS) and increase the opening frequency of chloride channels associated with these receptors, a mechanism resulting in CNS depression and sleep induction (43). The most common GABA_A_ receptor subtype in the brain is composed of two α and two β subunits, and one γ or δ subunit; the expression of different subunits on the receptor varies by location, physiological, pathological conditions of the brain and by treatment with different psychodrug (44).

BZDs differ from each other in their binding affinity with the GABA_A_ receptor, particularly in terms of binding affinity with the α subtypes. This explains why some BZDs are more suitable than others for certain indications. In particular, the various α receptor subtypes BZDs bind to mediate different functional effects: the α1 subtype is related to sedative effects, the α2 subtype relates to anxiolytic effects, the α3 subtype promotes myorelaxant and anxiolytic effects, and the α5 subtypes are memory modulators (44). The hypnotic effect of BZDs is induced by the simultaneous activation of the GABA_A_ receptor subtypes (containing the α1,2,3, and 5 subtypes) (44).

A further element that differentiates BZDs is their classification according to their elimination half-life: some BZDs have a very short half-life (less than 3 hours) or a short-to-intermediate half-life (6–12 hours) and others have a medium (intermediate) half-life (from 12−24 hours) or a long half-life (>24 hours) (45, 46). A cross-sectional study by Chen and colleagues found an association between the use of BZDs and sleep quality, which was dependent on the half-life of the BZD (47). Short-acting BZDs used only when required were associated with lower quality of night-time sleep and longer day-time napping than long-acting BZDs that were taken regularly (47).

BZDs are recommended for the short-term (≤4 weeks) treatment of insomnia (25). Clinical safety data are lacking to support their longer-term use (32, 38), which may be associated with the development of tolerance and dependence, and rebound insomnia after withdrawal (25). A recent network meta-analysis confirmed that BZDs improve sleep quality versus placebo, and as a class, were ranked as the best in subjective sleep quality compared with other pharmacological treatments for insomnia (32). A different network meta-analysis concluded that BZDs are effective for short-term use in insomnia, and that BZDs with intermediate half-lives such as lormetazepam and temazepam resulted in fewer discontinuations for any cause than BZDs with short or long half-lives, i.e. had better ‘acceptability’. Despite the limited data, long-term BZD use is common; one Spanish study reported in 2019 that 95.8% of all BZD prescriptions (in any indication, including insomnia) were being prescribed off-label as long-term medication (48). Longer term use is feasible, in some cases, by prescribing BZDs with a medium or short half-life, on an intermittent basis (25, 49, 50).

Since several BZDs are available, differing by onset and duration of action, adverse effects, and dosage requirements (25, 43), rational prescribing of BZDs requires an individualized risk-benefit evaluation. Physicians should carefully evaluate the patient’s chronotype, type of insomnia, age, and comorbidities against the specific drug’s characteristics and adverse effects (51). Physicians and patients should have a complete understanding of the potential adverse effects associated with BZD treatment, particularly from long-term treatment (see below), and especially in vulnerable patient populations, where dosage reduction is an essential safety precaution. Care should therefore be taken when prescribing BZDs in the following patient groups: elderly patients (because of the possible risk of falls/hip fractures and cognitive deficits), in patients with comorbid depression (because of a possible risk of suicide), in patients with alcohol misuse or who are taking medications for comorbidities that produce additive depressive effects on the CNS (e.g. antipsychotics, antidepressants, narcotic analgesics, anticonvulsants, anxiolytics and sedative antihistamines) (3). It is important to underline that the effects mentioned are possible side effects that depend on the patient’s susceptibility and do not represent a direct causal effect. However, they are minimized if the BZD is correctly dosed and the treated patient is clinically monitored. In addition, patients should understand other risks associated with BZDs, for example, the increased risk of misuse of opioids, particularly in patients who also use alcohol, and the potential for an increased risk of motor vehicle collisions due to adverse cognitive function effects, especially after long-term use (3).

While there is evidence of the long-term use of hypnotic drugs for primary chronic insomnia in clinical practice (52), United States (US) and European guidelines focus on reducing long-term BZD use due to the possible risk of tolerance and dependence, and favor the use of drugs with shorter half-lives (49, 50). In addition, European guidelines recommend intermittent dosing for patients under long-term treatment (25, 49). Intermittent use of BZDs has been shown to reduce risks associated with prolonged use (53).

Some researchers have postulated that intermittent prescribing of agents acting on the GABAergic system may help reduce the risk of dependence in chronic use (4). One study that compared a cohort of chronic BZD users with a matched cohort of intermittent BZD users showed evidence of significant excess risks associated with chronic compared with intermittent BZD use (4). Whether intermittent use would also reduce the risk of other adverse effects associated with long-term use of BZDs, especially in vulnerable patient populations, is not yet established.

### 5.2 Lormetazepam

Among the hypnotic BZDs, lormetazepam is a widely used agent that first became available in 1980, and is considered by some authors to be an effective and generally well tolerated BZD, with a low abuse liability (5). Indeed, lormetazepam is among the three most commonly prescribed hypnotic drugs (5). Lormetazepam is indicated for the short-term treatment (for a maximum of 4 weeks) of insomnia when it is disabling or subjecting the individual to extreme distress (54, 55).

Like other BZDs, lormetazepam enhances the action of GABA. Moreover, lormetazepam has specific pharmacodynamic and pharmacokinetic characteristics that make it consistent and preferable compared to other hypnotics. It has been shown to bind with high specificity and affinity to central BZD receptors (α1,2,3, and 5 subtypes) in *in vitro* radioreceptor binding assays (56). This explains the hypnotic and anxiolytic action of lormetazepam. As SAD is often associated with anxious components and evening hyperarousal (13), the action of lormetazepam on GABA_A_ receptors, reduces tension, anticipatory anxiety, and somatic overload in the evening, improving the onset of sleep, whereas Z-drugs do not have a significant anxiolytic effect, making them less suitable for patients who present both depressed mood and anxiety. Furthermore, with a terminal half-life of 8–12 hours, lormetazepam is considered to be a short/medium or intermediate-acting BZD (5) which is sufficient to ensure continuity of sleep without excessively prolonging morning sedation, whereas the Z-drugs have shorter half-lives (5, 57), and are effective for sleep induction but do not always ensure deep and stable sleep, leaving patients with SAD vulnerable to early awakenings or fragmented sleep. This means that when administered at the appropriate time (for example before 10–11 pm considering the sleep routine), low-dose (1 mg) lormetazepam has little or no residual effects on psychomotor performance and driving ability 12 hours after dose ingestion (58). The low incidence of hangover effects reported in a clinical study in patients with insomnia (59) is consistent with the lack of residual next-day effects reported by healthy volunteers administered repeated doses of lormetazepam 1 mg (60, 61).

Lormetazepam has a relatively limited impact on sleep structure (NREM/REM), promoting physiological sleep (62–64), whereas other hypnotics can significantly alter NREM or REM phases, worsening the perception of non-restorative sleep, a frequent phenomenon in SAD (63). These pharmacodynamic and pharmacokinetic properties explain the increased total sleep time and decreased wakefulness achieved with lormetazepam in healthy volunteers (65, 66). In addition, lormetazepam improved subjective ratings of sleep quality in healthy volunteers (61). Compared to molecules with a longer half-life (e.g., clonazepam, diazepam), lormetazepam offers a good balance between efficacy and rapid clearance, reducing residual morning drowsiness that can worsen the lethargy and low energy typical of SAD (64, 67).

Moreover, as with all BZDs, lormetazepam had a very low level of acute and chronic toxicity in animal toxicology studies (5). Lormetazepam is available as 0.5 mg and 1 mg tablets (54, 55), as well as an oral solution, for treating insomnia (68).

Comparative clinical studies involving patients with insomnia have shown that lormetazepam offers long-lasting action throughout the night, showing prolonged sleep latency, fewer night-time awakenings, improvements in subjective assessments of sleep parameters, and a sense of being “more refreshed” the next day compared with placebo (59, 69, 70). Improvements in sleep parameters were also observed in elderly patients (aged ≥65 years) with insomnia, when lormetazepam was given at a dose of 0.5 mg in combination with sleep hygiene training (versus sleep hygiene training alone) (62). In elderly patients with insomnia, lormetazepam induced sleep onset faster than zopiclone (71). In other clinical studies comparing lormetazepam with other BZDs in patients with insomnia and concomitant medical conditions, subjective sleep parameters were improved by a greater extent with lormetazepam than with diazepam, and adverse events were reported by none of the lormetazepam recipients versus 7.5% of diazepam recipients (72). Finally, in a comparison of lormetazepam (1 or 2 mg) and flurazepam (30 mg) in the treatment of insomnia, both drugs provided relief of insomnia versus placebo, but only the 2 mg dose of lormetazepam 2 mg was significantly better than placebo (73). In addition, when administered to inpatients with a major depressive episode without psychotic features, lormetazepam treatment significantly improved subjective sleep and decreased awakenings compared with placebo; lormetazepam had no effect on the severity of depression (74).

It is well known that there is a risk of abuse and dependence with all BZDs and Z-drugs (5, 57). To the best of our knowledge, no direct comparative studies on the incidence of dependence with lormetazepam versus other BZDs or Z-drugs after long-term use in patients with insomnia have been published. In the absence of comparative clinical data, it has been argued that the risk of dependence with lormetazepam is possibly lower than that of other benzodiazepines, although only for the oral tablet formulation of lormetazepam (5). It should be acknowledged that Italian data indicate a high rate of dependence associated specifically with an oral liquid formulation of lormetazepam (drops) available in Italy: 96.7–99.2% of patients abusing lormetazepam used the lormetazepam drops (75, 76). The preferential abuse of the drops versus oral tablets has been attributed to non-active constituents of the oral solution, such as alcohol, saccharine and flavoring, rather than to the active ingredient of lormetazepam (5). More recently, data from a French study indicated a lower proportion of patients abusing lormetazepam than several other BZDs (77). In this prospective study of 153 patients with alcohol use disorder hospitalized to manage alcohol withdrawal, BZD use was reported in 49% of patients and BZD misuse (abuse) in 18% of patients (36% of these patients were abusing BZDs). Among patients abusing BZDs, the most common BZD was diazepam (43.2%), followed by alprazolam (18.9%), whereas lormetazepam accounted for only 13.5% of abuse cases (77). Tolerance was identified in all patients abusing lormetazepam (77). These data underscore the importance of applying extreme caution in prescribing BZDs, including lormetazepam, in vulnerable patient populations, such as individuals with alcohol use disorder, as previously mentioned (see also section 5.2.1).

#### 5.2.1 Considerations for lormetazepam as a rational choice for insomnia in SAD

Seasonal Affective Disorder is often associated with dysregulation of the sleep–wake rhythm, leading to difficulties falling asleep, nocturnal awakenings, and daytime sleepiness with non-restorative sleep (1, 2, 13). Addressing sleep disturbances plays a clinically relevant role because sleep quality is closely intertwined with the severity of depressive symptoms, daytime energy, and the response to long-term treatments (phototherapy and antidepressants) (1, 2).

In light of the demonstrated clinical benefit of lormetazepam in patients with insomnia (69, 70, 72–74, 78), we speculated that this agent could be beneficial in the treatment of patients with insomnia associated with SAD. In this context, we would recommend that the dosage of lormetazepam should be adjusted based on individual chronotype, symptoms, and the severity of the sleep disorder. In this regard, it is crucially important to match the time of intake in relation to the patient’s chronotype to avoid not only morning residual effects but also night-time awakenings. Treatment duration should be as short as possible. The patient should be reassessed regularly, and the need for continued treatment should be carefully assessed. Treatment should be started with the lowest recommended dose, to be increased subsequently, taking care not to exceed the maximum dose. From the scant available data, only 2−3% of individuals taking BZDs might exceed the maximum permitted dose (79), representing a minority of patients, which is reassuring.

In winter SAD, there is often a tendency to sleep more, but in a less than restorative way, so lormetazepam can be used to improve the quality of sleep, generally with medium-low doses of 0.5−1.5 mg (68). In the case of summer SAD, more often associated with insomnia (difficulty falling asleep or staying asleep), the dosage may be slightly higher, i.e., 1−2 mg (68). Dosages should be re-evaluated in people with reduced hepatic, renal or respiratory function (54). Extreme caution is advised in people with a history of alcohol abuse or addictions (behavioral or substances) (54).

Lormetazepam can be used in either a cyclical or intermittent administration regimen; intermittent use may be particularly appropriate with respect to the seasonal course of the disorder. However, in the current absence of clinical protocols, studies are required to confirm whether lormetazepam used intermittently or cyclically is effective in treating SAD.

The total duration of treatment of lormetazepam ranges from a few days to 2–4 weeks, including the gradual withdrawal phase (54). In the opinion of the authors of this review, based on their extensive clinical experience, as for all BZDs and Z-Drugs, lormetazepam treatment should not be discontinued abruptly but withdrawn gradually, tapering by approximately 25% every 7 days.

### 5.3 Z-drugs

Z-drugs, together with BZDs, are among the most commonly prescribed hypnotics. These medications (including zolpidem, zopiclone, zaleplon, and eszopiclone) bind to the same recognition site as BZDs and are effective in improving parameters relating to sleep onset and sleep maintenance in patients with primary insomnia and co-morbid insomnia. However, unlike BZDs, zolpidem and eszopiclone have different pharmacodynamic (affinity/efficacy) effects at the GABA_A_ receptor subtypes. Accordingly, *in vitro* studies have shown that while the short-acting hypnotics triazolam and lormetazepam have almost the same affinity and efficacy for the GABA_A_ receptor subtypes containing α1,2,3, and 5 subunits, zolpidem has ten times greater affinity for the α1 subtype than for GABA_A_ receptors with α2 and α3 subunits and is devoid of action on the α5 receptor subtype (80). Moreover, eszopiclone, like zolpidem has a markedly lower affinity than triazolam and lormetazepam for all the receptor subtypes with α1,2,3, and 5 and much greater affinity for α5 than zolpidem, which per se does not bind to the α5 receptor subtype (81).

As for BZDs, chronic treatment with Z-drugs could be associated with the development of tolerance or dependence. Furthermore, the US Food and Drug Administration (FDA) has advised that rare but serious injuries have occurred with zolpidem due to sleep-related behaviors, including sleep-walking, sleep-driving, and engaging in other activities while not fully awake (82). The US FDA adds that these behaviors appear to be more common with zolpidem than with other prescription sleep medications, even when zolpidem is used at the lowest recommended dose.

## 6 Pragmatic perspectives

A critical reconsideration of the therapeutic class of BZDs is appropriate, as these drugs continue to suffer the stigma of the development of dependence and tolerance. With regard to the latter in particular, although the mechanism of onset is still unknown, one of the hypotheses underlying its development could be the modification, after prolonged use of high doses, in the gene expression of the receptor subunits of the GABA_A_ receptor (4, 81, 83, 84).

It is important to consider BZDs within a specific clinical context in which the use of short-term hypnotics could help fill an “unmet need”, i.e., that of assisting patients with SAD and insomnia. In fact, alternative treatment options such as antidepressants do not cure insomnia and are not appropriate for some patients due to potential adverse effects. In contrast, BZDs used in compliance with treatment schemes such as those described above can constitute a useful resource in the therapeutic armamentarium available to the physician. Indeed, the fifth edition of the Diagnostic and Statistical Manual of Mental Disorders (DSM-5-TR) (85) considers tolerance and dependence − very recurrent when dealing with the issue of abuse − as phenomena that do not occur when BZDs are taken under medical supervision.

Among the fears associated with the use of BZDs is a possible link between their long-term intake and the onset of neurological disorders, such as cognitive decline and dementia (86, 87), but the data for dementia are not definitive (87). Furthermore, the occurrence of anterograde amnesia has been highlighted as a class effect of BZDs (64). For example, in a placebo-controlled trial involving healthy male volunteers comparing lormetazepam (1 or 2 mg) with flunitrazepam (2 mg), anterograde memory impairments were the largest with flunitrazepam, less marked with lormetazepam 2 mg than with the 1 mg dose, while memory impairments with lormetazepam 1 mg did not differ from those with placebo (88). Even if anterograde amnesia were to occur with BZDs, its duration would be limited, given the short-to-intermediate half-life of the drug.

Clinicians should be correctly trained and educated in the use of BZDs. A recent qualitative investigation into the long-term use or prescription of BZDs has highlighted the fact that physicians lack resources, time, and specific knowledge about available treatments when addressing sleep- and anxiety-related problems in older-aged patients (89).

## 7 Conclusion

Sleep disorders, including insomnia, hypersomnia, and circadian rhythm disruptions, are common in SAD and can worsen emotional symptoms. Effective management often involves light therapy to regulate sleep-wake cycles, maintaining good sleep hygiene, and, if needed, medical interventions such as targeted medications or psychological therapies. Based on our current understanding of the pathophysiology of insomnia and SAD, we propose that hypnotic BZDs, such as lormetazepam, could be used to treat sleep disturbances in patients with SAD. In this context, lormetazepam represents a rational choice thanks to its balance between hypnotic efficacy, preservation of sleep architecture, control of evening anxiety, and limited risk of residual sedation. These elements make it preferable to hypnotics with an excessively short half-life (which do not sustain sleep) or an overly long half-life (which worsens the lethargy and low energy typical of SAD). Based on our clinical experience, short-term use of BZDs or BZDs on an intermittent dosing schedule may improve sleep quality and overall well-being in these patients. Nevertheless, well-controlled clinical trials in patients with SAD are required. We consider that our experience may serve as a stimulus for a critical reflection on the use of BZDs in SAD.
